# Uncommon presentation of craniospinal tuberculosis

**DOI:** 10.5339/qmj.2021.37

**Published:** 2021-09-06

**Authors:** Mohammed Abdulrabu, Ebrahim Ebrahim, Akram Warki, Ahmed Alsotuhy, Shahzad Anjum

**Affiliations:** ^1^Medical Education, Hamad Medical Corporation, Doha, Qatar E-mail: MAbdurabu@hamad.qa; ^2^Department of Emergency Medicine, Hamad Medical Corporation, Doha, Qatar; ^3^Department of Family Medicine, Hamad Medical Corporation, Doha, Qatar; ^4^Department of Radiology, Hamad Medical Corporation, Doha, Qatar

**Keywords:** craniospinal tuberculosis, uncommon presentation

## Abstract

Tuberculosis (TB) is a bacterial infection with multisystem presentations. Involvement of the central nervous system (CNS) is considered the most lethal form among all types. In addition to possible fatality, CNS TB has serious neurological sequelae. These morbidity issues along with diagnostic challenges doubles the clinical burden. In recent years, there have been improvements in diagnostic sensitivity and specificity due to advances in technology. Herein, we report an atypical case of a patient with TB who presented to our department and discuss the flow of the diagnostic workup.

## Introduction

Tuberculosis (TB) is a bacterial disease caused by aerobic acid-fast rods called *Mycobacterium tuberculosis* (*MTB*). *MTB* causes serious pulmonary and extrapulmonary systemic disease. TB is difficult to diagnose, particularly in resource-limited countries, because of the difficult, lengthy, and costly microbiological confirmation of the infection, leading to initiation of empirical treatment in some low-income endemic regions.^1,2^ This causes variability of epidemiological data on disease magnitude; however, the World Health Organization estimates that TB affects approximately 10 million people annually.^3^ Among all TB cases, TB of the central nervous system (CNS) accounts for 1% of all TB cases and for 5% of all extrapulmonary cases. Despite the rarity of this type of TB, it is the most serious type of systemic disease with high mortality, neurological complications, and disability.^4^ In this report, we describe the case of a man who presented with hallucination and paraplegia with magnetic resonance imaging (MRI) evidence of severe craniospinal leptomeningeal enhancement due to TB meningitis. To our knowledge, this is the first reported case of craniospinal TB and highlights important learning points that would enhance our understanding of this condition and improve patient care.

## Case Presentation

A 49-year-old married Indian man presented to the Emergency Department of Hamad General Hospital in Qatar, with fever, headache, lower limb weakness, and urinary incontinence for 1 month. His complaints were also associated with fluctuating episodes of confusion and agitation for 4 days. He had not experienced vomiting, convulsions, loss of consciousness, diplopia, facial weakness, or dysphagia. He had no history of trauma; fall; cardiac, respiratory, genitourinary, gastrointestinal, or musculoskeletal abnormality; or drug intake or intoxication. Review of his past medical documents revealed visits for evaluation of cervical lymphadenitis with inconclusive results. On assessment of his living conditions and housing, the patient stated that he lives in a crowded house with multiple roommates, of which one had prolonged cough without professional diagnosis.

On examination, the patient looked pale, had stable vital signs, agitated, confused, had neglected appearance with poor hygiene and having an odour smelling like urine. On neurological examination, he was conscious, alert, but not oriented, with a Glasgow coma scale of 12/15. Signs of meningeal irritation were negative. His motor examination showed decreased strength in both lower limbs (4/5) as well as diminished reflexes. His cranial nerves, sensory, and cerebellar examination were equivocal. Other systemic findings were normal.

His 1-month history of headache, fluctuating hallucinations, and confusion led us to consider the possibility of CNS infection. All basic laboratory tests were performed, including complete blood count, urea and electrolytes, blood culture and sensitivity, and C-reactive protein (CRP). Lumbar puncture was performed under conscious sedation. Initial investigations showed a minimal increase in leucocyte count and CRP, but he had normal renal and liver functions. Chest X-ray imaging did not show any abnormality, and computed tomography finding of the head was normal. High lymphocytes, high protein, and low glucose were detected in the cerebrospinal fluid (CSF).

**Figure 1.**
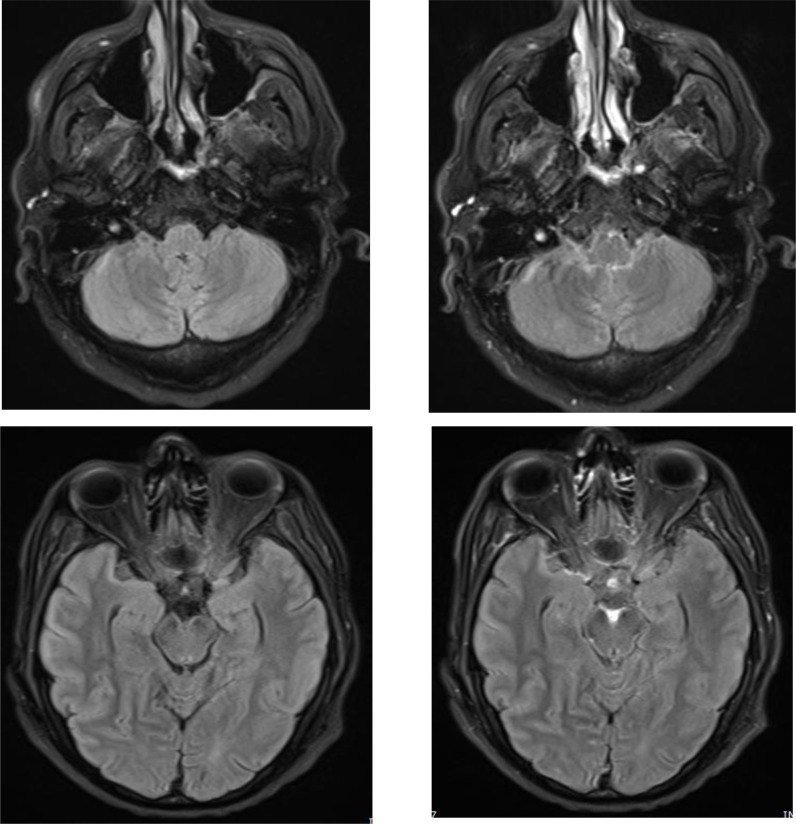
Axial FLAIR weighted images (WI) of the brain at the level of the posteriro fossa and temporal labes and after gadolinium based TV contrast injection showing basal meningeal thickening and enchancement predominantly circumscribing the brain stem structures and the interpeduncular cistern.

**Figure 2.**
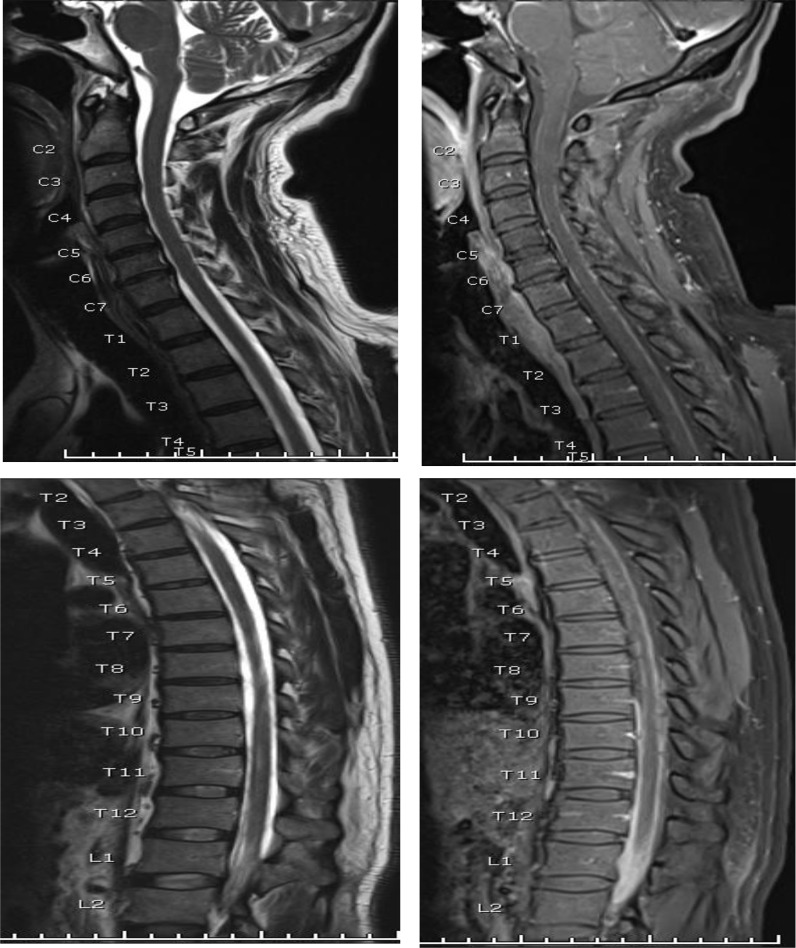
Sagittal tbl2WI and post-contract tbl2WI of the whole sping showing evident meningeal thickening and enchancement circumscribing the spinal cord and extending extensively along the cadua equina nerve roots almost completely replacing the CSF spaces and resulting in dry CSF puncutre.

**Figure 3.**
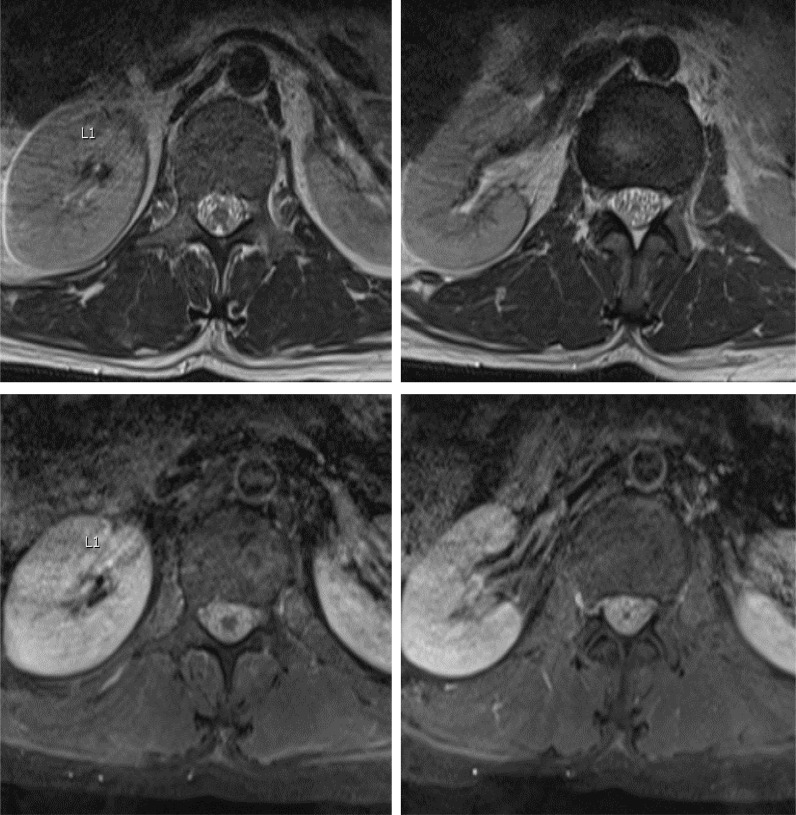
Axial tbl2 WI and tbl1 WI of the lumbar spine at the level of L1 and L2 vertebrae showing significant meningeal thickening and dense enhancement along the surface of the conus medullaries region and the cauda equina nerve roots replacing the CSF space at these levels.

The patient was admitted under the care of the medical team and was empirically started on anti-TB medications and antibiotics. MRI, acid-fast bacilli, and QuantiFERON gamma assay were performed. MRI of the head and spine (Figures 1–3) showed extensive basal intracranial meningeal thickening and enhancement extending along the spinal canal circumscribing the whole spinal cord down to the conus medullaris region (Figures 2, 3). Intramedullary high signal intensity was observed along the cervical spinal cord from C3 down to the C7 vertebral level and the lower thoracic spinal cord opposite the tbl10–11 and tbl12 vertebral level, yet no abnormal post-contrast enhancement was detected (2). This picture was highly suggestive of extensive craniospinal TB meningitis.

The patient showed dramatic improvements in his orientation and conscious level after 7 days of receiving anti-TB management and was discharged from the acute medical ward to the rehabilitation center to address his lower limb weakness.

## Discussion

TB meningitis is the most lethal clinical presentation of MTB infection, with a mortality rate that may be exceptionally high (6%–65%, average 33%), despite treatment with anti-TB chemotherapy.^5^ It is also responsible for severe disability in many survivors.^6^

We encountered a relatively rare case of TB meningitis in an adult patient, which manifested as fever, lower limb weakness, and urine incontinence along with fluctuating confusion and agitation in the absence of elevated inflammatory markers, neck stiffness, and photophobia, which are characteristic of meningitis.

Although head CT results were normal, MRI of the head and spine showed extensive craniospinal involvement consistent with TB. These findings are very rare, and reviewing existing literature showed no reports related or resembling our case, which makes it unique in comparison with other cases, e.g., the case encountered in Japan of a rare TB meningitis in a patient with spinal tumor but without spinal involvement.^7^

To diagnose this case, following meticulous history taking, which was oblivious to his condition, physical examination was initiated, which showed decreased power and reflexes in the lower limb with hypertonia and hyperreflexia in the upper limb. These findings were unusual in comparison with reports in previous studies that most cases of TB meningitis present with fever, meningeal signs, and altered mental state.^8^

As regards diagnostic modalities, basic laboratory tests were initiated, but did not show anything significant or specific. CT of the head revealed oddly normal result; therefore, lumbar puncture was performed, showing high lymphocytes, high protein, and low glucose, which are consistent with TB meningitis.^9^ However, CSF and blood cultures were negative for TB. Negative cultures are common in TB meningitis, as revealed in previous reports that <  20% of cases have MTB detected in CSF cultures.^2^

TB was confirmed by MRI of the patient's head and spine, which showed meningeal thickening and enhancement from his brain until the conus medullaris (Figures 1–3). MRI has been gaining robust diagnostic power in diagnosing cranial and intraspinal TB. This case support the finding of Gupta et al., that TB can be diagnosed by MRI.^9^

Polymerase chain reaction (PCR) for TB was also performed, but with negative result. However, a repeat test of the sample showed a positive result, which is common, owing to the ability of PCR test to detect certain and specific genes that are difficult to detect. Advances in molecular TB diagnostics in the last decade have led to the development of TB tests that are highly accurate and faster than conventional microbiological tests; emerging technologies promise to continue this trend. Moreover, nucleic acid amplification tests (NAATs) are exerting a positive clinical and probably epidemiological impact. However, for long-term outcomes, such as mortality, the effect of NAAT is more ambiguous.^10–12^

Our patient was started on anti-TB medications because of his history of contacting a sick person who also lived in the same house. The patient showed dramatic improvements in mental state, but admission to a rehabilitation facility was necessary to address his lower limb weakness and bladder control caused by the extensive lesions.^13^ The patient was not a candidate for surgical intervention because of the dramatic improvement gained with medical treatment. In general, many patients with TB will develop some kind of disability.^14^

In conclusion, the significance of this case report is related to the unusual presentation of the case. In the presented case, TB affects the head and spine of an otherwise immunocompetent individual. This report also features the importance of MRI in diagnosing patients who present with altered mental state and lower limb weakness and in identifying findings in the brain, spinal cord, and leptomeninges. The study also reinforces the necessity of regular follow-up of TB cases to limit both mortality and disability.

### Ethical approval

Informed consent and approval of the patient was obtained prior to drafting the case report.
